# Effectiveness of the Assessment of Burden of Chronic Obstructive Pulmonary Disease (ABC) tool: study protocol of a cluster randomised trial in primary and secondary care

**DOI:** 10.1186/1471-2466-14-131

**Published:** 2014-08-07

**Authors:** Annerika HM Slok, Johannes CCM in ’t Veen, Niels H Chavannes, Thys van der Molen, Maureen PMH Rutten-van Mölken, Huib AM Kerstjens, Guus M Asijee, Philippe L Salomé, Sebastiaan Holverda, Richard PN Dekhuijzen, Denise Schuiten, Gerard van Breukelen, Daniel Kotz, Onno CP van Schayck

**Affiliations:** 1 Department of Family Medicine, Maastricht University, CAPHRI School for Public Health and Primary Care, PO Box 616, 6200 MD Maastricht, The Netherlands; 2 Department of Pulmonology, Sint Franciscus Gasthuis, Rotterdam, The Netherlands; 3 Department of Public Health and Primary Care, Leiden University Medical Centre, PO Box 9600, 2300 RC Leiden, The Netherlands; 4 Department of Primary Care, University of Groningen, University Medical Centre Groningen, Antonius Deusinglaan 1, 9713 AV Groningen, The Netherlands; 5 Groningen Research Institute for Asthma and COPD (GRIAC), University Medical Centre, Groningen, The Netherlands; 6 Erasmus University Rotterdam, Institute for Health Policy and Management/Institute for Medical Technology Assessment, PO Box 1738, 3000 DR Rotterdam, The Netherlands; 7 Department of Pulmonary Diseases, University of Groningen, University Medical Centre Groningen, Antonius Deusinglaan 1, 9713 AV Groningen, The Netherlands; 8 PICASSO foundation for COPD, Alkmaar, The Netherlands; 9 Huisartsenzorg IJsselstein, locatie ’t Steyn, IJsselstein, The Netherlands; 10 Lung Foundation Netherlands, Amersfoort, The Netherlands; 11 Department of Pulmonary Diseases, University Medical Centre Nijmegen, Nijmegen, The Netherlands; 12 Department of Methodology & Statistics, Maastricht University, CAPHRI School for Public Health and Primary Care, PO Box 616, 6200 MD Maastricht, The Netherlands

**Keywords:** Chronic obstructive pulmonary disease, Disease burden, Quality of life, Patient reported outcomes, Shared decision making, GPs, Pulmonologists, Patient empowerment

## Abstract

**Background:**

Chronic Obstructive Pulmonary Disease (COPD) is a growing worldwide problem that imposes a great burden on the daily life of patients. Since there is no cure, the goal of treating COPD is to maintain or improve quality of life. We have developed a new tool, the Assessment of Burden of COPD (ABC) tool, to assess and visualize the integrated health status of patients with COPD, and to provide patients and healthcare providers with a treatment algorithm. This tool may be used during consultations to monitor the burden of COPD and to adjust treatment if necessary. The aim of the current study is to analyse the effectiveness of the ABC tool compared with usual care on health related quality of life among COPD patients over a period of 18 months.

**Methods/Design:**

A cluster randomised controlled trial will be conducted in COPD patients in both primary and secondary care throughout the Netherlands. An intervention group, receiving care based on the ABC tool, will be compared with a control group receiving usual care. The primary outcome will be the change in score on a disease-specific-quality-of-life questionnaire, the Saint George Respiratory Questionnaire. Secondary outcomes will be a different questionnaire (the COPD Assessment Test), lung function and number of exacerbations. During the 18 months follow-up, seven measurements will be conducted, including a baseline and final measurement. Patients will receive questionnaires to be completed at home. Additional data, such as number of exacerbations, will be recorded by the patients’ healthcare providers. A total of 360 patients will be recruited by 40 general practitioners and 20 pulmonologists. Additionally, a process evaluation will be performed among patients and healthcare providers.

**Discussion:**

The new ABC tool complies with the 2014 Global Initiative for Chronic Obstructive Lung Disease guidelines, which describe the necessity to classify patients on both their airway obstruction and a comprehensive symptom assessment. It has been developed to classify patients, but also to provide visual insight into the burden of COPD and to provide treatment advice.

**Trial registration:**

Netherlands Trial Register, NTR3788.

## Background

The prevalence of Chronic Obstructive Pulmonary Disease (COPD) is increasing globally due to an aging population and continued exposure to risk factors [1]. COPD has a major impact on the lives of patients’. The prevalence of COPD is expected to increase, and is projected to be the fourth leading cause of death and the seventh leading cause of disability-adjusted life years (DALYs) lost worldwide by 2030 [2,3].

Since there is no cure for the disease, the goal of COPD treatment is to maintain or improve patients’ quality of life. As such, the care of patients with COPD is currently shifting towards a more patient-centred model. More attention is paid to the impact of the disease on the patient’s life [1]. It is therefore necessary to focus on more than just airway obstruction, as measured by the forced expiratory volume in one second (FEV1), which has a poor correlation with many patient reported outcomes in COPD [4]. Health status measurements have been propagated as an important part of managing COPD, both in primary and secondary care [5]. Previous studies showed that a poor health status is a predictor for hospitalization and mortality [6-8].

**Figure 1:**
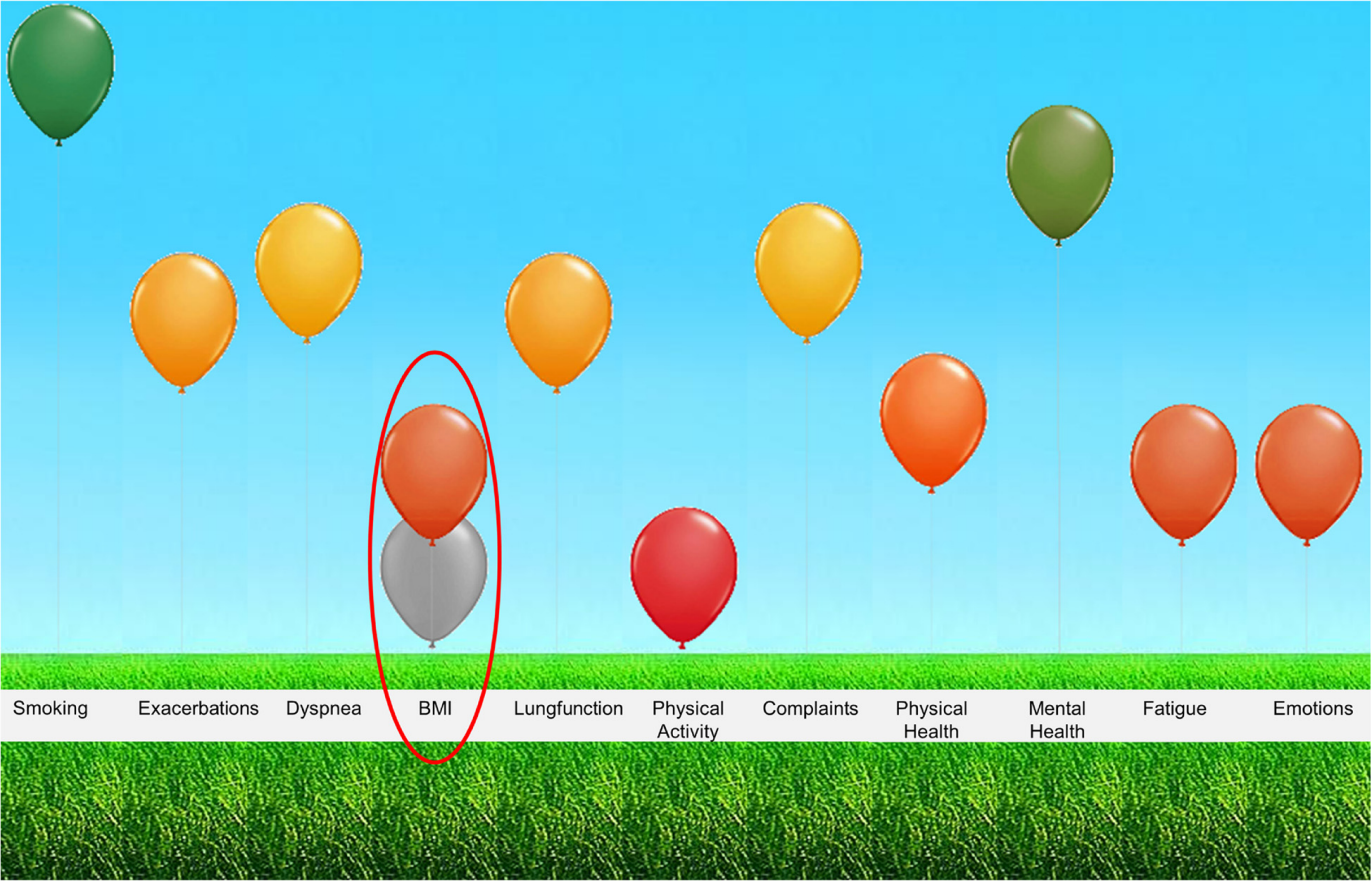
Visualization of the dimensions influencing integrated health status (Assessment of Burden of COPD tool), changed after treatment.

Health status questionnaires can also be used to determine the burden of COPD as experienced by patients. However, current health status questionnaires do not fully cover patients’ experienced burden of disease [9]. Hence, we developed a new tool, the Assessment of Burden of COPD (ABC) tool, which consists of three elements. The first element is the ABC scale, a 14-item questionnaire that measures five different domains of the burden of disease as experienced by the patient: symptoms, functional state, mental state, emotional state, and fatigue. The second element comprises a number of additional indicators: smoking status, exacerbation history, dyspnoea, body mass index (BMI), lung function and physical activity. When combined with the ABC scale an overall assessment of a patient’s integrated health status is obtained. The third element is a computer program used to visualize the scores on all items into green, orange and red balloons and link treatment advice to these balloons (see Figure 1) [9].

The purpose of the ABC tool is to assess the experienced burden of disease and to provide both patient and healthcare provider with a visual overview of the individual patient’s burden of COPD. The tool is designed to facilitate shared decision making, in which patients and healthcare providers are both responsible for the focus of treatment , selecting the corresponding treatment options, formulating a personal goal and make a tailored treatment plan [10]. Patients need to be encouraged to take the lead in their own treatment, and the ABC tool aims to facilitate in this process. It can be used during consultations to monitor the patient’s integrated health status, including the experienced burden of COPD, and to treat the patient accordingly.

The development of the tool has been described elsewhere [9].

The reliability, validity and responsiveness of the ABC scale, the first element and core component of the ABC tool, still needs to be assessed. The scale is based mainly on the Clinical COPD Questionnaire (CCQ) [11], so we assume that it is a valid and reliable instrument. However, to be thorough we will analyse the internal consistency and test-retest reliability. Furthermore, we will analyse the convergent validity, divergent validity, known-group differences and responsiveness.

Since the effectiveness of the ABC tool has not yet been tested, we will conduct a cluster randomised controlled trial (RCT). The trial will take place in both primary and secondary care settings, to include patients with varying disease severity. We aim to evaluate the effectiveness by answering the following research question:

In COPD patients, varying in disease severity, does application of the ABC-tool increases the percentage of patients with a clinically relevant improvement on the Saint George Respiratory Questionnaire (SGRQ) [12], between baseline and 18 months follow-up, as compared with usual care?

## Methods/Design

### Study design

**Figure 2:**
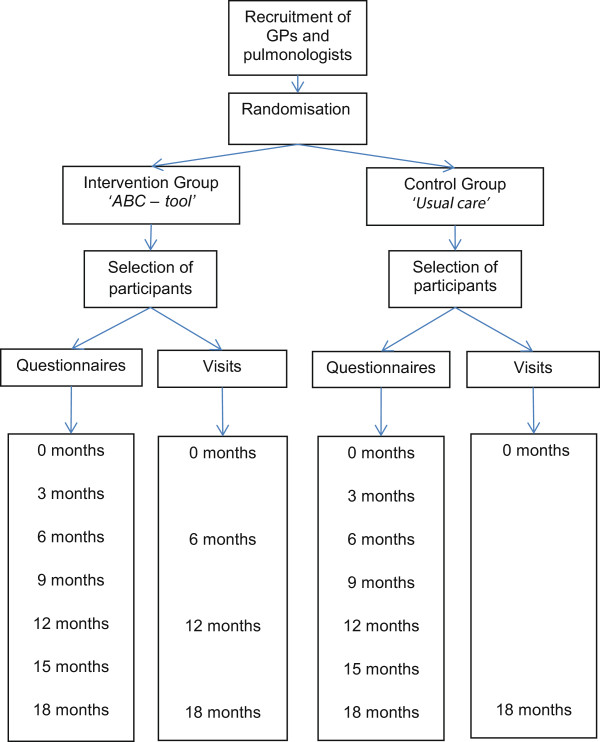
Flow of the study.

The study is a two-armed cluster RCT that compares an intervention group, using the ABC tool with a control group receiving usual care. The duration of follow-up period is 18 months. During this period there are seven measurements, including a baseline and final measurement. The flow of the study is presented in Figure 2.

This study has been approved by the Medical Ethics Committee of the Atrium-Orbis-Zuyd Hospital in the Netherlands.

### Selection, recruitment and randomisation

This study will take place in general practices and hospitals throughout the Netherlands. The researchers will contact general practitioners (GPs) and pulmonologists using a GP network (CAHAG; COPD and Asthma GP Advice Group) and the pulmonologists association (NVALT; Dutch Association of Physicians for Lung Diseases and Tuberculosis). To avoid contamination of care (i.e. patients from the control group receiving the intervention because they are treated by the same caregiver), it is vital that the randomisation between the groups takes place at caregiver-level and not at patient-level. Therefore, healthcare providers will be randomly assigned to intervention or control group in a 1:1 ratio with the help of a computer program designed by the Maastricht University Centre for Data and Information Management (MEMIC). Randomisation will be stratified by type of caregiver (GP or pulmonologist) and variable blocks of two, four or six will be used to make sure there are an equal number of caregivers from primary and secondary care in the intervention and control group. The researcher will enter the names of the healthcare providers into the computer program, which will then randomly allocate them to either the intervention or control group.

Participating healthcare providers will be asked to invite patients from their listed patient databases to participate in the trial. Patients that meet the inclusion criteria and exclusion criteria (see next paragraph) will be sent an information letter including an informed consent form. Healthcare providers will also directly invite patients to participate if they see them during consultations. If the patient agrees to participate in the study, an appointment will be made with the healthcare provider to discuss further details of the trial, recheck whether the patient is eligible for the study and sign the informed consent form.

### Inclusion and exclusion criteria

The inclusion criteria will be: a confirmed diagnosis of COPD (post-bronchodilator FEV1/Forced Vital Capacity < 0.7) by spirometer, age 40 years or above, and the ability to understand and read the Dutch language. The exclusion criteria will be: an exacerbation less than six weeks ago, hard-drug addiction, life-threatening co-morbid conditions, and current pregnancy. Exacerbations are defined as an acute event characterized by worsening of a patient’s respiratory symptoms beyond normal day-to-day variation that necessitates prednisolone and/or hospital admissions for COPD.

### Intervention group

The actual intervention will be the use of the ABC tool by patients and healthcare providers during routine consultations. Patients will fill out the ABC scale, which measures the patient’s experienced burden of COPD, in the waiting room (without supervision) [9]. The healthcare provider will then enter the results of the ABC scale into a computer program and add the results of additional items (i.e. smoking status, exacerbation history, dyspnoea, body mass index (BMI), lung function and physical activity). These items and the ABC scale together will show the integrated health status of the patient, illustrated by a visual representation with coloured balloons. The balloons represent all of the patient’s health status items, including the different domains of the ABC scale (see Figure 1). A high, green balloon means that the patient scores well on a particular item/domain; an orange balloon means an intermediate score; and a low, red balloon means that the patients scores poorly on that item/domain. Patients and healthcare providers will be prompted to discuss the balloons together, according to the principles of shared decision making (SDM) [13], and will be advised to work on the items represented by the red and orange balloons. These represent the items with possible room for improvement if the right treatment is provided. Each balloon can be clicked on, showing a pop-up with treatment advice.

The algorithm behind the ABC tool was developed based on a previous treatment algorithm attached to the CCQ [14], and optimized for this study according to national and international guidelines [15-17]. The cut-off points are based on the current Dutch COPD Healthcare Standard guidelines [18], and experts’ experiences. The level of the balloon was determined by using a continuous scale to grade the displayed value. The treatment recommendations were written by pulmonologists and GPs based on the current national COPD guidelines. Two versions of the algorithm were written, one for the use in primary care, based on the standard of the Dutch College of General Practitioners [15] and the other for the use in secondary care, based on the NVALT guidelines [16]. The treatment advice was written during a number of meetings and groups discussions, and the algorithm was tested extensively by healthcare providers with different backgrounds throughout the Netherlands. Examples of the treatment advice are provided in Additional file 1.According to the principles of shared decision making, patients and healthcare providers will be advised to discuss the possible treatment options within the patient’s possibilities. One or more pieces of advice can be selected and will automatically and electronically be placed in the patient’s written treatment plan. During the consultation, healthcare providers should guide patients in setting a personalized treatment goal. This personal goal should be written down as an achievable goal, in the patient’s own words. The treatment plan aims to achieve the personal goal. At each follow-up moment, the intervention group will complete the ABC scale and use the ABC tool. The tool will show both the current balloons and the balloons from the previous consultation, displayed in grey (Figure 1). The tool can be used to monitor a patient’s integrated health status over time and adjust the treatment as necessary.

### Control group

The ABC scale and tool (including its software) will only be provided in the intervention group. The patients in the control group will receive care as usual and their healthcare providers will not be instructed by the research team.

### Measurements

#### ***Intervention group***

Patients in the intervention group will receive individualized treatment guided by their integrated health status, as measured predominantly by the ABC scale. Patients will visit their healthcare providers at least four times during the 18 months follow-up. During these consultations, healthcare providers will record lung function, number of exacerbations in the previous year and since previous visit, smoking behaviour, BMI and comorbidity. Additional file 2 shows all items of the integrated health status. Questionnaires will be sent to patients’ homes at 0, 3, 6, 9, 12, 15 and 18 months (see Table 1).

**Table 1:** Measuring moments

	**Time (months)**						
**Completed by patients at the GP practice or hospital**	**0**	**3**	**6**	**9**	**12**	**15**	**18**
Assessment of burden of COPD scale*	x		x		x		x
Medical Research Council*	x		x		x		x
Physical activity*	x		x		x		x
**Completed by patients at home**							
Demographics	x						x
The Saint George Respiratory Questionnaire	x		x		x		x
COPD Assessment Test	x		x		x		x
EuroQol5d-5 level Questionnaire	x		x				x
Patients Assessment Chronic Illness Care questionnaire	x				x		x
Healthcare use, medication use	x	x	x	x	x	x	x
Employment status, absence from paid work	x	x	x	x	x	x	x

*Completed by patients in the intervention group only.

#### ***Control group***

Patients in the control group will receive care as usual. The patients will visit their healthcare provider for the baseline measurement (0 months) and the 18-month follow up. At these consultations lung function and BMI will be measured, and smoking status, comorbidity, number of exacerbations in the previous year and since previous visit will be monitored. Questionnaires will be sent to patients’ homes at 0, 3, 6, 9, 12, 15 and 18 months (see Table 1).

### Outcomes

#### ***Primary outcome***

The primary outcome measure will be the proportion of patients in the intervention group with a clinically meaningful improvement on the SGRQ [19], that is, a decrease of at least 4 points between baseline and 18 month follow-up, compared with the control group.

The SGRQ consists of 50 questions with scores that range from 0 (best) to 100 (worst). Higher scores indicate more advanced impairment in health-related quality of life [12].

#### ***Secondary outcomes***

1. The net proportion of patients in the intervention group with a clinically meaningful change of 4 points on the SGRQ between baseline and 18 month follow-up [19], compared with the control group. The net proportion will be calculated by subtracting the percentage of patients improved by 4 points on the SGRQ from the percentage of patients decreased by 4 points.

2. The difference in the proportion of patients from the intervention group and control group with a clinically meaningful improvement of at least 4 points on the SGRQ (a decrease in the SGRQ score), between baseline and 6 months and between baseline and 12 months follow-up.

3. The absolute difference SGRQ score at 18-month follow-up between the intervention group and control group.

4. Health related quality of life will also be measured by the COPD Assessment Test (CAT) [20]. Differences on total scores will be calculated between baseline and 12 months follow-up and between baseline and 18 months follow-up. The CAT is an eight item questionnaire with scores ranging from 0 to 40, with higher scores indicating greater impairment of health related quality of life [20].

Differences in number of exacerbations and lung function, as recorded by the healthcare providers, will be calculated between the two groups, with respect to the number of exacerbations at a given time point and to change over time.

5. An economic evaluation will be performed at 6 and 12 months. This evaluation will compare differences in costs with differences in effects (Cost Effectiveness Analysis) and quality adjusted life years (Cost Utility Analysis).

Questionnaires will be used to record visits to physicians and healthcare providers, medication use, hospital admissions, employment status and absence from paid work every three months. For the economic evaluation, costs will be calculated from a healthcare perspective and a societal perspective.

The EuroQol5d-5 level Questionnaire (EQ-5d-5 L) measures the generic health-related quality of life. The EQ-5D-5 L consists of 5 dimensions to describe health - mobility, self-care, every day activities, pain/discomfort and anxiety/depression - each with five levels of functioning (e.g. no problems, slight problems, moderate problems, severe problems, unable to/severe problems) [21]. The domain descriptions are combined with population-based values to derive a health utility index [22]. In addition to the descriptive system and the off-the-shelf value sets, the EQ-5D-5 L includes a visual analogue scale (VAS) that individuals can use to rate their own health on a scale from 0 (worst imaginable health) to 100 (best imaginable health).

6. The process evaluation will consist of two parts: patients’ evaluation of the level of integrated healthcare will be measured at baseline, 12 months and the final visit (18 months), using the Patients Assessment Chronic Illness Care questionnaire (PACIC) [23]. The PACIC is a 20 item questionnaire, with a five point response scale (ranging from 1 = ‘almost never’ to 5 = ‘almost always’). Higher scores represent a more frequent presence of the aspect of structured chronic care. Furthermore, treatment plans (recorded in the ABC tool) will be compared qualitatively with the delivered care to patients (as reported by the patients).

A process evaluation among healthcare providers includes assessing factors that may have hindered or facilitated the implementation of the ABC tool in the management of COPD care. This evaluation, among approximately 10 to 15 participating healthcare providers, will be performed by means of semi-structured group interviews and individual interviews. Questions that will be discussed include; “How easy is it to use the ABC-tool?”, “How did patients react on the visual display?”, and “What barriers did you encounter when using the ABC scale and the ABC tool?”. The methods for the process evaluation will be described elsewhere in more detail.

### Sample size calculation

The required sample size of 360 patients (180 patients per arm) is based on the following assumptions:

1) A clinical response (a clinically meaningful improvement of at least 4 points [19]) of 50% in the treated arm versus 30% in the control arm [24,25] (implying an effect size *d* = 0.42 for the clinical response), and a power of 80% to detect a difference in the clinical response rate of the primary outcome between the treated and control arm with a two-tailed alpha of 5%. This assumption gives a sample size of 180 patients in total (90 patients per arm), ignoring at first the design effect due to clustering of patients within physicians.

2) The number of participating GPs will be about twice as large as the number of pulmonologists.

3) An estimated availability of 5 patients per GP and 8 patients per pulmonologist on average. This, together with assumptions 1 and 2, gives a total of 20 GPs and 10 pulmonologists. However, the following three steps result in a sample size which is twice as large, that is 40 GPs and 20 pulmonologists.

4) An intraclass correlation coefficient (ICC) of 0.05, meaning that about 5% of the total outcome variation within each arm is between GPs and between pulmonologists, instead of between patients of the same physician. Literature suggests that an ICC of 0.05 is a good default value for trials in primary care [26-28]. Combined with assumptions 2 and 3, and allowing for 10% more clusters (healthcare providers) to compensate the power loss due to variation in cluster size, that is, in number of patients included per healthcare provider, this ICC of 0.05 implies a design effect of 1.38 [29]. The number of clusters must thus be multiplied with 1.38.

5) A dropout rate of 25% of patients and/or clusters, to be compensated by multiplying the number of clusters to be included by 1.33 (since 75% of 1.33 is 1). Dropouts will be included into the analyses (intention to treat), but contribute less to the power due to missing data, hence the present correction.

6) Data analysis of the primary outcome with the recommended PQL2 (penalized quasi-likelihood) estimation method which requires a further multiplication of the number of clusters with a factor of 1.10 [30].

Combining assumptions 4, 5 and 6 gives a multiplication factor of 1.38 * 1.33 * 1.10 = 2 for the number of GPs and pulmonologists as computed in steps 1 to 3, leading to the planned sample size of 40 GPs, 20 pulmonologists and 360 patients in total.

### Statistical analysis

All baseline variables and outcomes will be summarized with descriptive tables and, in the case of repeated measures, plotted against time per treatment arm.

The primary and first two secondary outcomes are binary outcomes and will be analysed with mixed logistic regression, using PQL2 or numerical integration as method of estimation.

All quantitative outcomes will be analysed with mixed linear regression, with physician, patient and measurement as three levels at which sampling error occurs, and as predictors treatment arm, time point of measurement, treatment by time interaction, patient demographics and other prognostic variables. Generalized linear mixed models with negative binomial distribution will be used to estimate the adjusted difference in exacerbations. Costs will also be analysed with generalized linear mixed models using a log-normal distribution or gamma distribution. A p-value of < .05 will be considered statistically significant.

All data will be analysed according to the intention-to-treat principle, including all randomised healthcare providers and patients. To prevent missing data, patients will receive reminders for the questionnaires if they do not return the questionnaire within three weeks. If necessary, multiple imputation to cope with missing values will be used [31].

## Discussion

This paper describes the design of a cluster RCT to evaluate the effectiveness of the newly developed ABC tool. The development of the tool was based on the insight that the severity of COPD has to be determined by both the severity of the airway obstruction and the burden of disease. The 2014 guidelines of the Global Initiative for Chronic Obstructive Lung Disease (GOLD), recommend a comprehensive symptom assessment in determining the severity of COPD [17]. The guidelines also introduced a combined COPD assessment resulting in an ABCD classification. This novel classification integrates symptom severity as measured by mMRC [32], CCQ [11] of CAT [20], versus prognostic indicators of pathophysiologic severity assessed by airflow obstruction and exacerbation history [33]. The ABC tool offers a different operationalization of this new approach and goes beyond classification. The strength of the tool is that it provides a profile of scores on many different domains of COPD-related health. The tool not only focuses on the quantification of the burden of COPD, but determines the actual integrated health status. In addition, it provides a treatment algorithm which gives the patient and healthcare provider insight into treatment options accordingly. The tool offers a visual display of the integrated health status using balloons to show the scores on the different domains of the ABC scale and additional items such as smoking status and exacerbation history. Furthermore, the algorithm provides treatment advice, including pharmacologic options as well as non-pharmacologic options, to formulate a personalized treatment goal and treatment plan. The tool is designed to provide insight into the patient’s individual burden of COPD and therefore enables to make a tailored treatment plan and to make the patient feel co-responsible for their own treatment, using principles of shared decision making [13].

An important issue during the process of designing the study was the selection of an appropriate primary outcome measure. It is more common to use objective parameters, such as the airway obstruction, as primary outcomes [34]. However, airway obstruction is hypothesized to correlate poorly with health status and/or quality-of-life [4]. We therefore decided to use a health-related quality-of-life questionnaire. Since treatment in the intervention arm is based on the outcomes of this new instrument, which is based on the CCQ, a different quality-of-life instrument will be used to measure the primary outcome. We decided to use the SGRQ [12], a disease-specific quality-of-life instrument. We hypothesize that the use of the ABC tool in daily care will have a positive effect on the quality-of-life of COPD patients As the CAT is part of the assessment according to the GOLD guidelines [17], we decided to use it as a secondary outcome measure.

The trial will be conducted throughout the Netherlands, in both primary and secondary care. This will make it possible to evaluate the effectiveness of the ABC tool on the health status of patients with different COPD severities. Conducting the study in many clusters throughout the Netherlands might lead to a higher external validity of the results. Furthermore, it might help facilitating the process of implementation. If the results of this trial are as expected, it will be advised to implement the ABC tool in routine care. To facilitate this, it is recommended to adopt the tool in the registration systems of healthcare providers and in healthcare standards. Using the tool in routine care provides structure in each consultation, and more insight for patients in all factors related to COPD. It might help healthcare providers to discuss difficult issues with their patients related to COPD, such as emotions, smoking cessation and impact of COPD on daily life. Furthermore, it might lead to more commitment of the patient and taking the lead in their own treatment, increasing their self-management.

## Abbreviations

ABC scale/tool: Assessment of Burden of COPD scale/tool; CAT: COPD Assessment Test; CCQ: Clinical COPD Questionnaire; COPD: Chronic Obstructive Pulmonary Disease; DALY: Disability Adjusted Life Years; EQ5d-5 L: EuroQol5d-5 level Questionnaire; FEV1: Forced Expiratory Volume in one second; GP: General Practitioner; mMRC: modified Medical Research Council; NVALT: in Dutch: Nederlandse Vereniging van Artsen voor Longziekten en Tuberculose; PACIC: Patients Assessment Chronic Illness Care questionnaire; PQL: Penalized Quasi-Likelihood; RCT: Randomised Controlled Trial; SDM: Shared Decision Making; SGRQ: The Saint George Respiratory Questionnaire; VAS: Visual Analogue Scale.

## Competing interests

The authors AS, JCCMi’tV, NC, HAMK, SH, PS, DS, GvB, DK and GMA declare that they have no conflicts of interest in relation to this article.

OS received several unrestricted institutional grants from Pfizer, Boehringer Ingelheim, Novartis, AstraZeneca and GlaxoSmithKline.

TvdM developed the CCQ, received grants, reimbursement for travel and fee’s for speaking and advisory boards from AstraZeneca, GlaxoSmithKline, Boehringer Ingelheim, Novartis, Teva and MSD.

The Erasmus University, Institute for Medical Technology Assessment, where MRM is employed, has received funding for designing and conducting cost-effectiveness studies of COPD drugs from multiple pharmaceutical companies (Boehringer Ingelheim, Nycomed, Pfizer). MRM has received speaker fees and compensation for serving on advisory boards for GSK, Boehringer Ingelheim, Pfizer, Nycomed and Novartis. MRM does not own stock of any pharmaceutical company.

PNRD has received reimbursements for attending symposia, fees for speaking, organising educational events, funds for research or fees for consulting from AstraZeneca, Boehringer-Ingelheim, Chiesi, Merck Sharp & Dohme, Mundipharma, Novartis, Takeda, Almirall and Teva.

## Authors’ contributions

All authors were responsible for the development of the ABC tool and developing the research protocol. AS and OS were responsible for drafting the manuscript. JCCMi’tV, NC, TvdM, MRM, HAMK, PS, SH, PNRD, DS, DK, GvB and GMA made critical revisions to the manuscript. AS and OS reached consensus on the final version for submission. All authors read and approved the final manuscript. OS had the final responsibility for the content.

## Pre-publication history

The pre-publication history for this paper can be accessed here:

http://www.biomedcentral.com/1471-2466/14/131/prepub

## Supplementary Material

Additional file 1 Examples of treatment advice in the algorithm. Click here for file

Additional file 2 Integrated Health Status. Click here for file
