# Supplementary material for: Effectiveness of the Assessment of Burden of Chronic Obstructive Pulmonary Disease (ABC) tool: study protocol of a cluster randomised trial in primary and secondary care
Source: BMC Pulm Med. 2014 Aug 7;14:131. doi: 10.1186/1471-2466-14-131 (PMC4130125; doi:10.1186/1471-2466-14-131)
Supplement: Additional file 1 — Examples of treatment advice in the algorithm. [file 1471-2466-14-131-S1.docx]

**Additional file 1 Examples of treatment advice in the algorithm**

| **Symptoms** | **Symptoms (subscore > 1)**  The total score on the CCQ is….  The score of the questions about symptoms is…..    *Treatment advice for health care provider (select if necessary):*   - Provide patient with information about COPD. - Check adherence / inhalation-techniques. If necessary, provide explanation/demonstration. - Check if inhalation therapy (medication + type of inhaler) is correct for this patient. Amend, if necessary. - If necessary start short-acting bronchodilators or add a second   (long-acting) bronchodilator.    **Symptoms (subscore>2):**  The total score on the CCQ is…..  The score of the questions about symptoms is...  *Treatment advice for health care provider (select if necessary):*   - Provide patient with information about COPD. - Referral to secondary care, in accordance with the health care standard.   Re-entering primary care:   - Continue policy secondary care |
| --- | --- |
| **Functional state** | **Functional state (subscore>1):**  The total score on the CCQ is…..  The score of the questions about physical functioning is...  *Treatment advices for health care provider (select if necessary):*   - Provide patient with information about COPD. - Provide patient with advice about physical activity/exercise.     **Functional state (subscore>2):**  The total score on the CCQ is…..  The score of the questions about physical functioning is...  *Treatment advice for health care provider (select if necessary):*   - Provide patient with information about COPD. - Referral to secondary care, in accordance with the health care standard.   Re-entering primary care:   - Continue following secondary care policy. |
| **Mental state** | **Mental state (subscore>1):**  The total score on the CCQ is…..  The score of the questions about the mental state is...  *Treatment advice for health care provider (select if necessary):*   - Provide patient with information about COPD. - Discuss mental problems.     **Mental state (subscore>2):**  The total score on the CCQ is…..  The score of the questions about the mental state is...  *Treatment advice for health care provider (select if necessary):*   - Provide patient with information about COPD. - Referral to secondary care, in accordance with the health care standard.   Re-entering primary care:   - Continue policy secondary care. |
| **Fatigue:**  **poor > 2** | You indicate that you suffer from fatigue.  *Treatment advice for health care provider (select if necessary):*   - Treat non-pulmonary causes of fatigue. - Consider referral to secondary care if pulmonary cause is the only possible indication. - Consider referral to psychologist/psychosocial care. - Consider referral to support groups / Luchtpunt. |
| **Emotions:**  **poor >2** | You indicate that you have emotional problems.  *Treatment advice for health care provider (select if necessary):*   - Treat non-pulmonary causes of emotional problems. - Consider referral to secondary care if pulmonary cause is the only possible indication. - Consider referral to psychologist/psychosocial care. - Consider referral to support groups / Luchtpunt. |
| **Lung function:**  **<50%** | Your FEV1 score is < 50%.  *Treatment advice for health care provider (select if necessary):*   - Did the patient have a flu shot? If not, emphasize the importance. - Referral to secondary care, in accordance with the health care standard.   Re-entering primary care:   - Check and implement treatment advice provided in secondary care. - Has FEV1 decreased in comparison to previous measurements? In case of rapid deterioration (> 100ml per year), refer to pulmonologist, in accordance with the health care standard. |
| **Weight loss:**  **>10%** | Since previous visit, you have lost……  Check whether weight loss is related to COPD, or whether there is another explanation.  *Treatment advice for health care provider (select if necessary):*   - Dietary advice in primary care. - Additionally, referral to secondary care for further analyses of the weight loss, in accordance with the health care standard.   Re-entering primary care:   - Check and implement treatment advice provided in secondary care. - Consider referral to dietician. |
| **Exacerbations:**  **2 or more** | In the past year, you have had two exacerbations. It is important to evaluate the determinants of these temporary worsening’s.  *Treatment advice for health care provider (select if necessary):*   - Check adherence / inhalation-techniques. If necessary, provide explanation/demonstration. - Check if inhalation therapy (medication + type of inhaler) is correct for this patient. Amend, if necessary. - Did the patient have a flu shot? If not, emphasize the importance. - Assessment of other possible causes of recurrent exacerbations. - Add ICS, if not yet in use. - Make follow-up appointment with practice nurse for exacerbation treatment plan.   Already ICS:   - Refer to secondary care, in accordance with the health care standard.   Follow up advice:   - Check up after 4-6 weeks   Persisting FEV1 < 50 % (post) of predicted, refer to secondary care in accordance with the health care standard. |
| **Smoking:**  **Yes** | You smoke. It is very important for COPD-patients to quit smoking.  Are you motivated to quit smoking? Yes / No  *Treatment advice for health care provider (select if necessary):*  When patient is motivated to quit smoking:   - Provide smoking cessation advice. - Make follow-up appointment with practice nurse for smoking cessation counselling, in accordance with the health care standard. - Refer to smoking cessation clinic.   When patient is not yet motivated to quit smoking:   - Provide smoking cessation advice. - Consider referral to websites and provide information about smoking cessation. - Consider motivational sessions to guide the patient towards a quit attempt. |
