# Supplementary material for: Effectiveness of the Assessment of Burden of Chronic Obstructive Pulmonary Disease (ABC) tool: study protocol of a cluster randomised trial in primary and secondary care
Source: BMC Pulm Med. 2014 Aug 7;14:131. doi: 10.1186/1471-2466-14-131 (PMC4130125; doi:10.1186/1471-2466-14-131)
Supplement: Additional file 2 — Integrated Health Status. [file 1471-2466-14-131-S2.docx]

**Additional file 2 Integrated Health Status**

| **On average, during the past week, how often did you feel:** | | | | | | | | | |
| --- | --- | --- | --- | --- | --- | --- | --- | --- | --- |
|  | Never | | | Hardly ever | A few times | Several times | Many times | A great many times | Almost all the time |
| **1** Short of breath at rest? | ☐ | | | ☐ | ☐ | ☐ | ☐ | ☐ | ☐ |
| **2**  Short of breath doing physical activities? | ☐ | | | ☐ | ☐ | ☐ | ☐ | ☐ | ☐ |
| **3** Concerned about getting a cold or your   breathing getting worse? | ☐ | | | ☐ | ☐ | ☐ | ☐ | ☐ | ☐ |
| **4** Depressed (down) because of your breathing   problems? | ☐ | | | ☐ | ☐ | ☐ | ☐ | ☐ | ☐ |
| **In general, during the past week, how much of the time:** | | | | | | | | | |
|  | | Never | | Hardly ever | A few times | Several times | Many times | A great many times | Almost all the time |
| **5** Did you cough? | | ☐ | | ☐ | ☐ | ☐ | ☐ | ☐ | ☐ |
| **6** Did you produce phlegm? | | ☐ | | ☐ | ☐ | ☐ | ☐ | ☐ | ☐ |
| **On average, during the past week, how limited were you in these activities because of your breathing problems:** | | | | | | | | | |
|  | | | Not limited at all | Very slightly limited | Slightly limited | Moderately limited | Very limited | Extremely limited | Totally limited/ or unable to do |
| **7** Strenuous physical activities (such as climbing stairs, hurrying, doing sports)? | | | ☐ | ☐ | ☐ | ☐ | ☐ | ☐ | ☐ |
| **8** Moderate physical activities (such as walking,   house work, carrying things)? | | | ☐ | ☐ | ☐ | ☐ | ☐ | ☐ | ☐ |
| **9**  Daily activities at home (such as dressing,   washing yourself)? | | | ☐ | ☐ | ☐ | ☐ | ☐ | ☐ | ☐ |
| **10** Social activities (such as talking, being with children, visiting friends/relatives)? | | | ☐ | ☐ | ☐ | ☐ | ☐ | ☐ | ☐ |
| **How often in the past week did you suffer from:** | | | | | | | | | |
|  | | | Never | Hardly ever | A few times | Several times | Many times | A great many times | Almost all the time |
| **11** Worry? | | | ☐ | ☐ | ☐ | ☐ | ☐ | ☐ | ☐ |
| **12** Listlessness? | | | ☐ | ☐ | ☐ | ☐ | ☐ | ☐ | ☐ |
| **13** A tense feeling? | | | ☐ | ☐ | ☐ | ☐ | ☐ | ☐ | ☐ |
| **14** Fatigue? | | | ☐ | ☐ | ☐ | ☐ | ☐ | ☐ | ☐ |

Patients will complete questions 1 to16.

Health care providers will report questions 17 to 25

15. MRC dyspnoea grades:

1. “I am not suffering from breathlessness”;
2. “I only get breathless with strenuous exercise”;
3. “I get short of breath when hurrying on the level or up a slight hill”;
4. “I walk slower than people of the same age on the level because of breathlessness or have to stop for breath when walking at my own pace on the level”;
5. “I stop for breath after walking 100 meters or after a few minutes on the level”;
6. “ I am too breathless to leave the house”.
7. Do you exercise enough according to the Dutch standard (at least 5 days a week, 30 minutes of moderate intensive exercise)?
   - Yes
   - No
8. Smoking status of the patient
9. Number of exacerbations in the previous year
10. Number of exacerbations since previous visit
11. Weight (in kilograms)
12. Height (in centimetres)
13. Relevant comorbidity (asthma, depression, diabetes mellitus, heart failure, malignancies, osteoporosis, other….)
14. FEV1 in ml
15. FEV1%
16. FER

| **Domains of the ABC scale** | **Questions** |
| --- | --- |
| Symptoms | 1, 2, 5, 6 |
| Functional State | 7, 8, 9, 10 |
| Mental State | 3, 4 |
| Emotions | 11, 12, 13 |
| Fatigue | 14 |
